# Inflammatory cytokine levels correlate with amyloid load in transgenic mouse models of Alzheimer's disease

**DOI:** 10.1186/1742-2094-2-9

**Published:** 2005-03-11

**Authors:** Nikunj S Patel, Daniel Paris, Venkatarajan Mathura, Amita N Quadros, Fiona C Crawford, Michael J Mullan

**Affiliations:** 1 Roskamp Institute, 2040 Whitfield Avenue, Sarasota, FL34243, USA

## Abstract

**Background:**

Inflammation is believed to play an important role in the pathology of Alzheimer's disease (AD) and cytokine production is a key pathologic event in the progression of inflammatory cascades. The current study characterizes the cytokine expression profile in the brain of two transgenic mouse models of AD (TgAPPsw and PS1/APPsw) and explores the correlations between cytokine production and the level of soluble and insoluble forms of Aβ.

**Methods:**

Organotypic brain slice cultures from 15-month-old mice (TgAPPsw, PS1/APPsw and control littermates) were established and multiple cytokine levels were analyzed using the Bio-plex multiple cytokine assay system. Soluble and insoluble forms of Aβ were quantified and Aβ-cytokine relationships were analyzed.

**Results:**

Compared to control littermates, transgenic mice showed a significant increase in the following pro-inflammatory cytokines: TNF-α, IL-6, IL-12p40, IL-1β, IL-1α and GM-CSF. TNF-α, IL-6, IL-1α and GM-CSF showed a sequential increase from control to TgAPPsw to PS1/APPsw suggesting that the amplitude of this cytokine response is dependent on brain Aβ levels, since PS1/APPsw mouse brains accumulate more Aβ than TgAPPsw mouse brains. Quantification of Aβ levels in the same slices showed a wide range of Aβ soluble:insoluble ratio values across TgAPPsw and PS1/APPsw brain slices. Aβ-cytokine correlations revealed significant relationships between Aβ1–40, 1–42 (both soluble and insoluble) and all the above cytokines that changed in the brain slices.

**Conclusion:**

Our data confirm that the brains of transgenic APPsw and PS1/APPsw mice are under an active inflammatory stress, and that the levels of particular cytokines may be directly related to the amount of soluble and insoluble Aβ present in the brain suggesting that pathological accumulation of Aβ is a key driver of the neuroinflammatory response.

## Background

Alzheimer's disease is a progressive neurodegenerative disorder characterized by intra-cellular abnormally phosphorylated tau protein and extra-cellular beta amyloid plaques. It has been suggested that inflammation may be a key player in the pathophysiology of AD as evidenced by epidemiological studies which have revealed that the long term use of non-steroidal anti-inflammatory drugs reduces the risk of developing AD [1-3]. Transgenic mouse models of Alzheimer's disease that over-express β-amyloid (Aβ) exhibit significant cerebrovascular inflammation and microgliosis around areas of plaque deposition [4-7]. Chronic administration of ibuprofen can reduce plaque pathology and brain Aβ levels in these animal models of AD [8,9].

There are numerous reports of increased levels of cytokines in the brains of Alzheimer's disease patients, and in transgenic mouse models of Alzheimer's disease [10-12]. However, all these reports have focused on a small number of cytokines within the same sample. It is not clear which cytokines are key in promoting and maintaining the inflammatory environment in the AD brain. Furthermore, it is unclear which Aβ species (1–40, 1–42, soluble or insoluble) are most closely related to cytokine levels. Multiplex technology enables the simultaneous quantification of many cytokines within a single sample.

By examining different mouse models of AD using multiplex technology, it is possible to more clearly characterize the particular cytokines which maintain the inflammatory environment and to relate them to particular forms of Aβ (1–40, 1–42, soluble or insoluble).

There is considerable debate over which length of Aβ and which conformations are most potently toxic. Recently, specific oligomeric forms have been shown to be most toxic to neurons. These soluble species of Aβ differ from the higher-molecular-weight aggregated insoluble forms that are found precipitated in the AD patient and mouse brain. This study sought to determine whether soluble or insoluble Aβ fractions were most closely related to cytokine levels.

## Materials and methods

### Organotypic brain slice cultures

Mouse brain slice cultures were prepared as previously described [29]. Briefly, 15-month-old PS1 (M146L), TgAPPsw (K670M / N671L), PS1/APPsw and wildtype littermates were humanely euthanized and the brains extracted under sterile conditions. One-mm-thick brain slices were sectioned from co-ordinates 1 to -4 from bregma using a mouse brain slicer. Sections were cultured in neurobasal medium with 5% B27 supplement (Gibco-Invitrogen, CA) and Penicillin-Streptomycin-Fungizone mixture (Cambrex Corp., NJ). After 40 hours, media was collected for quantification of cytokine levels.

Multi-plex cytokine array analysis was performed using the Bio-plex protein multi-array system, which utilizes Luminex-based technology [13]. For the current experiments, a mouse 12-plex assay was used according to the recommendations of the manufacturer (BioRad, CA).

### Measurement of Aβ levels in brain slices

Brain slices were washed with PBS (BioSource, CA), and 300 μl of lysis buffer was added. Lysis buffer consisted of mammalian protein extraction reagent (Pierce-Endogen, IL) with 1X protease inhibitor cocktail XI (Calbiochem, CA), 100 μM Sodium Orthovanadate, and 1 μM Phenylmethylsulfonyl Fluoride (PMSF) (Sigma-Aldrich, MO). The resulting mixture was sonicated using a sonic dismembrator (Fisher Scientific, PA)

Protein content in each slice was determined using the bicinchoninic acid (BCA) protein reagent kit (Pierce-Endogen, IL), as per the manufacturers protocol. Insoluble Aβ was extracted using 70% formic acid as previously published [14].

Aβ content in brain slices was determined using human Aβ 1–40 and Aβ 1–42 ELISA detection kits (Biosource, CA), as per the manufacturers protocol.

### Statistical analyses

For statistical analyses, ANOVA and t-tests were performed where appropriate using SPSS for Windows release 10.1. Hierarchical cluster analysis of Aβ-cytokine data from brain slices were performed with the R program . A correlation matrix was constructed using the raw data and subsequently converted to a distance matrix by subtracting each element in the correlation matrix from 1. The distance matrix was used as the dissimilarity matrix for building an hierarchical cluster using the averaging method. The resulting dendrogram consists of closely related members under the same node. The farther one needs to traverse across the tree to reach another member, the higher the dissimilarity represented. The distance from the base in the y-axis represents dissimilarity or 1-r, where r is the correlation co-efficient.

## Results

### Cytokine production by organotypic brain slice cultures

**Figure 1:**
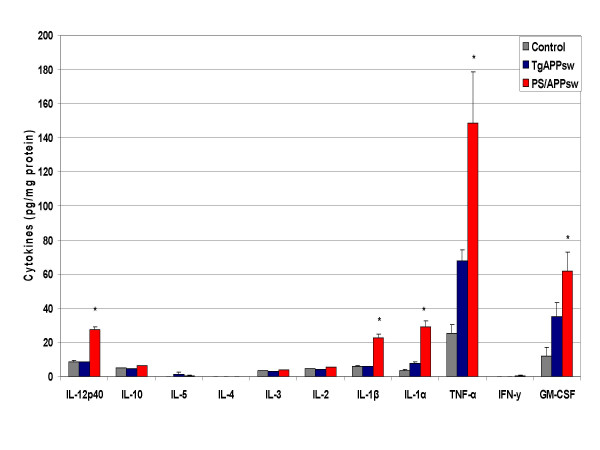
**Cytokine production by brain slices from transgenic mouse models of AD at 15 months of age. **Freshly harvested brain slices were incubated in neurobasal medium with B27 supplement. Media was collected after 24 hours, and cytokine levels measured. Mean concentrations (N = 15) +/- standard error are expressed in picograms per milligram of protein. P < 0.05 was considered statistically significant.

**Figure 2:**
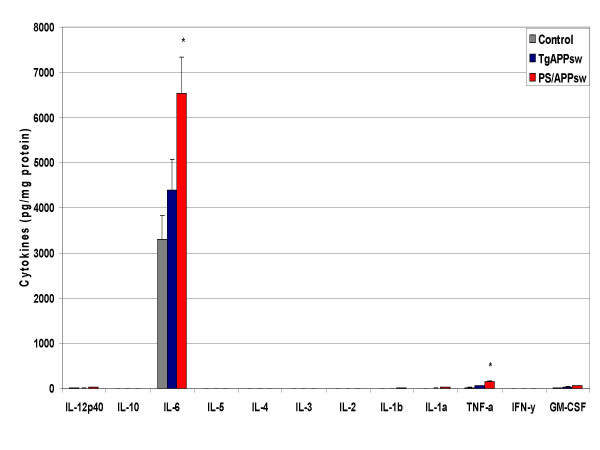
**Cytokine production by brain slices from transgenic mouse models of AD at 15 months of age. **Freshly harvested brain slices were incubated in neurobasal medium with B27 supplement. Media was collected after 24 hours, and cytokine levels measured. Mean concentrations (N = 15) +/- standard error are expressed in picograms per milligram of protein. P < 0.05 was considered statistically significant.

Cytokine production was evaluated by multi-plex cytokine array analysis using the cell culture supernatant of organotypic brain slice cultures from control, PS1 (Presenilin 1 mutant heterozygotes), TgAPPsw, and TgPS1/APPsw mice at 15 months of age. We chose non-transgenic littermates as controls for the TgAPPsw mice and the PS1 animals as controls for the PS1/APPsw mice as the PS1 animals were the littermates of the PS1/APPsw mice. There were no significant differences in cytokine production between control slices and PS1 slices showing that PS1 over-expression does not directly induce inflammatory events. Compared to control slices, production of IL-1α, TNF-α, GM-CSF and IL-6 was increased in TgAPPsw slices (figs. 1, 2). Compared to TgAPPsw slices, PS1/APPsw brain slices produced significantly more IL-12p40, IL-1β, IL-1α, TNF-α, GM-CSF and IL-6. Across control, TgAPPsw, and PS1/APP transgenic brain slices, there was a graduated increase in IL-1α, TNF-α, GM-CSF and IL-6.

### Correlation between Aβ level and cytokine production by transgenic mouse brain slices

Quantification of amyloid levels in brain mouse slices revealed that PS1/APPsw mice produce significantly more total Aβ as compared to TgAPPsw mice at the same age, and levels of insoluble and soluble Aβ (both 1–40 and 1–42) correlated well with each other (Table 1). Analysis of the ratio of soluble:insoluble Aβ revealed a wide range of values across the TgAPPsw and PS1/APPsw mouse brain slices, with a 15.3-fold variance for Aβ 1–40 and a 5.4-fold variance for Aβ 1–42 (for Aβ 1–40, comparison of soluble:insoluble ratios revealed an average difference of 3.9 fold, and an average 1.7-fold difference for Aβ 1–42).

**Table 1:** Quantification of Aβ levels in TgAPPsw and PS1/APPsw mouse brain slices. Data expressed as picograms/mg protein, mean ± S.E.M. for 13 determinations.

	TgAPPsw	PS1/APPsw
Soluble Aβ1–40	331.15 ± 35.36	4957.79 ± 322.30
Soluble Aβ1–42	68.11 ± 6.82	1644.29 ± 90.30
Insoluble Aβ1–40	67619.38 ± 7089.61	4095442 ± 409212.3
Insoluble Aβ1–42	6837.22 ± 2741.70	286463.3 ± 31395.63

**Figure 3:**
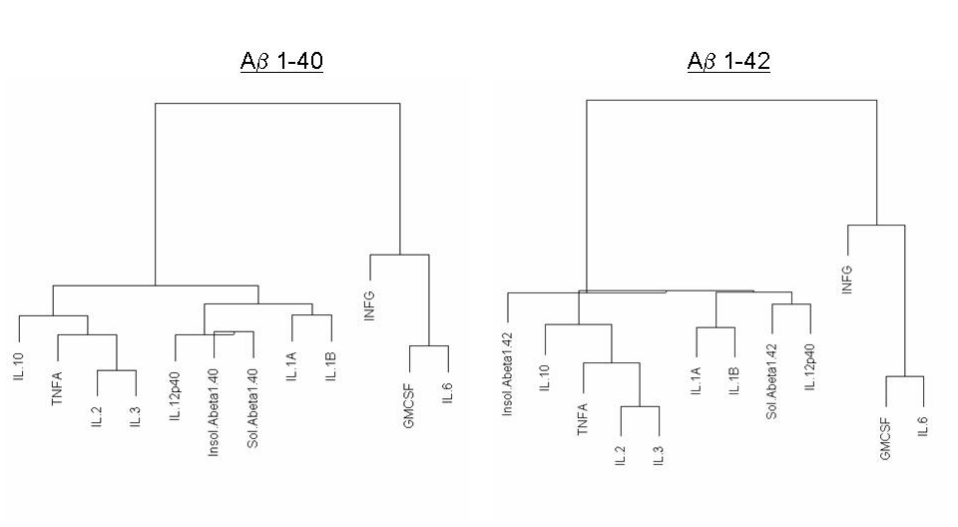
**Dendrogram correlations of Aβ1–40 and Aβ1–42-cytokine relationships. **Closely related members appear under the same node. The farther one needs to travel across the tree to reach another member, the greater the dissimilarity.

**Figure 4:**
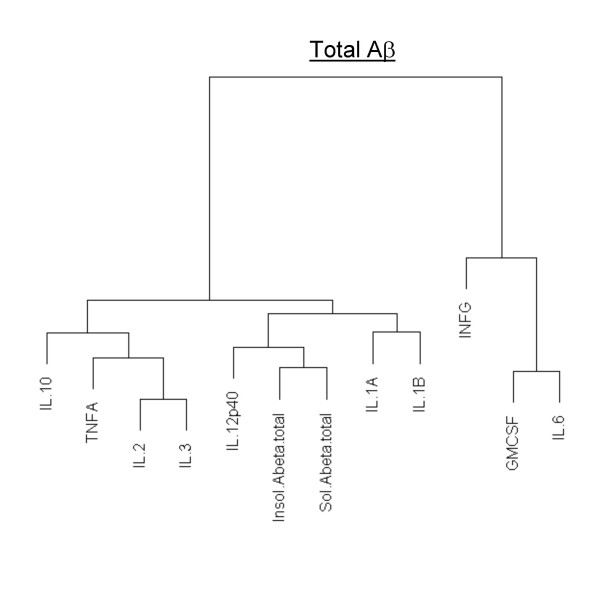
**Dendrogram correlations of Total Aβ (Aβ1–40+Aβ1–42)-cytokine relationships. **Closely related members appear under the same node. Total Aβ levels were calculated by adding soluble and formic acid extracted Aβ. The farther one needs to travel across the tree to reach another member, the greater the dissimilarity.

**Figure 5:**
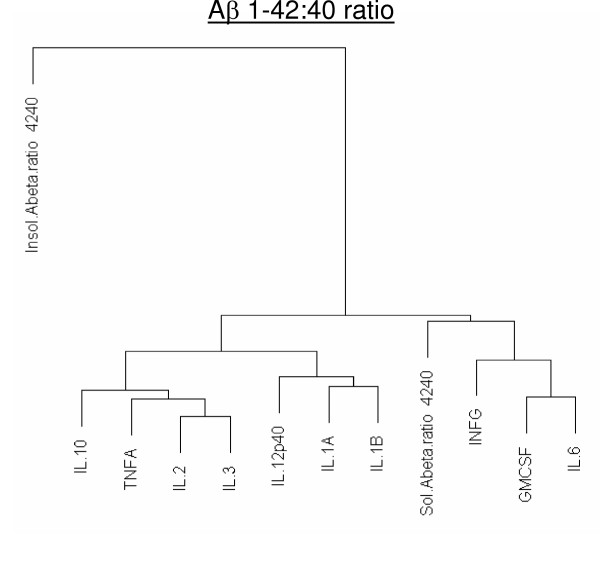
**Dendrogram correlations of (Aβ1–42:40 ratio)-cytokine relationships. **Total Aβ1–42:40 ratio's were calculated for both soluble and formic acid extracted Aβ. Closely related members appear under the same node. The farther one needs to travel across the tree to reach another member, the greater the dissimilarity.

Although all the cytokines that changed in the transgenic brain slices were correlated with increases in Aβ levels, some showed a closer relationship than others to Aβ levels (Figs. 3, 4, and 5). A table of r-correlation values is given in Additional file 1. It is important to note that the dendrograms depict the closeness of a correlation between a particular cytokine and Aβ levels, and that all the members in the dendrograms are in fact highly correlated with Aβ levels (1% significance was considered as r >= 0.496, and 5% significance was considered as r >= 0.388). IL-4 and IL-5 were not produced in detectable amounts, were therefore omitted from the dendrograms. Of all the cytokines, IL-12p40 showed the strongest correlation with levels of both Aβ1–40 and 42 (soluble or insoluble). IL-1α and IL-1β were also highly correlated with Aβ1–40 and 42 (soluble or insoluble).

## Discussion

Levels of both peripheral and local CNS cytokines are elevated in AD patients, indicating that there is cellular activation occurring in response to inflammatory stimuli [15-20]. However, there is still considerable debate over exactly what is triggering this inflammation. Studies using mouse models of AD have shown that ibuprofen is effective in reducing plaque pathology and also in improving behavioral deficits characteristic of these transgenic models [8,21]. The transgenic mouse models used to study AD exhibit some of the pathological features seen in the AD patient brain and show an increased production of inflammatory markers such as COX-2, PGE_2 _and also increased levels of the pro-inflammatory cytokines IFN-γ and IL-12, TNF-α, IL-1α, IL-1β and IL-6 [12,22]. Pathological analysis of tissue from AD patients and from mouse models of AD shows that there is extensive astrocytic and microglial activation around areas of Aβ plaque deposition [6,7]. In addition, the chronic use of non-steroidal anti-inflammatory drugs (NSAIDs) has been associated with a reduced risk of developing AD [23,24], suggesting that inflammation is an important contributor to the pathophysiology of AD.

One aim of this study was to create a cytokine expression profile for organotypic brain slice cultures from transgenic mouse models of Alzheimer's disease, and to further relate this increase to the level of Aβ present in the brain. Another purpose of our study was to determine whether inflammatory events may be correlated with the accumulation of particular forms of Aβ; either soluble or insoluble.

In the current study, we used the organotypic brain slice culture model to assess multiple cytokine production in the culture medium surrounding brain slices from transgenic mice that are engineered to over-produce Aβ. Cytokine production from 15-month-old control, PS1, TgAPPsw and PS1/APPsw mouse brain slices was assessed using the Bioplex cytokine multi-array system. Cytokine levels were not significantly elevated in PS1 brain slices compared to control slices, indicating that the PS1 (M146L) mutation does not have a significant impact on cytokine production. No significant change in the production of IL-4 and IL-10 was observed in the brains of these transgenic mice compared to their respective controls, indicating the absence of an anti-inflammatory response. All of the cytokines that were increased in the TgAPPsw brain slices (IL-1α, TNF-α, GM-CSF and IL-6) were further increased in the PS1/APP brain slices. This suggests that the presence of these inflammatory molecules is related to the amount of β-amyloid protein present, in agreement with a pro-inflammatory effect of Aβ [25-29]. A recent report has also shown increases in IL-1β, IL-6 and TNFα in-vivo after intra-cerebral administration of fibrillar Aβ into rat brain [30].

In order to further understand the correlation between the amount of Aβ and cytokine levels in the brains of transgenic mice, levels of both soluble and insoluble (formic acid-extracted) Aβ1–40 and 1–42 were quantified in the same slices from which cytokine production was measured, allowing a direct correlation of Aβ-cytokine levels.

Levels of soluble and insoluble Aβ1–40 correlated well with each other, and the same was observed for Aβ1–42. As expected, quantification of Aβ levels generally revealed significantly higher amyloid levels in the PS1/APPsw mouse brain slices compared to TgAPPsw (for soluble Aβ, approximately 15 fold more Aβ1–40, and 20 fold more 1–42) but there was considerable slice-to-slice variation in soluble and insoluble Aβ levels within and between genotypes. The TgAPPsw and PS1/APPsw mice express equal levels of the APPsw molecule, but the PS1/APPsw model produces greater levels of Aβ and develops plaques at an earlier age (10 weeks) [31-33]. This increased deposition of Aβ in the PS1/APPsw mouse is due to a PS1 mutation, resulting in increased production of Aβ1–42 [34-36].

The Aβ data in the current report found a significant range of values for soluble:insoluble Aβ ratios between brain slices. This broad spread of values allowed correlation with equally wide ranges of cytokine production. This approach of examining Aβ-cytokine correlations within the same slices in the same aged animals eliminated the confounding factor of age related changes in cytokine production. Both Aβ1–40 and 1–42 correlated closely with all the cytokines that changed in the brain slices, but the correlation was particularly striking with IL-12p40. IL-12 is a hetero-dimeric cytokine which can comprise two subunits; IL-12p40 and IL-12p35. It is produced mainly by monocytes and macrophages and is a crucial factor in directing the T-cell response to infection, by inducing a Th1-type cytokine response. Our data agrees with that of previous reports showing that IL-12p40 is strongly up-regulated in-vitro (in response to an inflammatory stimulus) and in-vivo in the cerebral cortex of TgAPPsw mice [12,37,38].

IL-1, which was increased in the transgenic brain slices, is a major immune-response molecule functioning in the periphery and brain. The family comprises three related proteins (IL-1α, IL-1β and IL-1 receptor antagonist (IL-1ra)). IL-1α and IL-1β are two different isoforms of IL-1 that have similar affinities for their receptor IL-1R, and therefore have similar activities. Both are capable of inducing inflammatory cascades in-vivo and in-vitro, and it has been shown that they are capable of up-regulating expression of astrocyte-derived S100B and APP [39,40]. It has been shown that IL-1β can promote β-secretase cleavage of APP in human astrocytes and thereby increase production of Aβ1–40 and 1–42 [41,42]. It is also known that accumulation of plaques and the formation of neurofibrillary tangles are correlated with increased IL-1 levels in the AD brain [43-45]. Certain polymorphisms of IL-1A (the gene for IL-1α) are associated with late onset AD, although there is controversy as to whether all IL-1 gene polymorphisms represent risk factors for AD [46-50]. Microglia, in particular, have been shown to locally up regulate IL-1α at both the protein and mRNA level when inflamed, a situation that occurs in chronic disease states such as AD [51]. Both IL-1α and IL-1β can enhance the translation of APP mRNA in human astrocytes [52]; an up-regulation of IL-1α/β production in-vivo could therefore increase Aβ production, and an inflammatory cycle with increased Aβ levels may further increase IL-1α/β production.

The Aβ 1–42:40 ratio is also of considerable interest in relation to cytokine levels and although there are currently no studies correlating Aβ 1–42:40 ratio with cytokine levels in-vivo, certain reports have suggested that cytokines can modulate Aβ production [53-55]. PS1 mutations are known to cause a shift in the production of Aβ species, favoring the production of Aβ1–42 over 1–40 and causing an increase in the Aβ1–42:40 ratio [56]. Since TNF-α correlated better with the level of Aβ1–42 than with that of Aβ 1–40, and correlated particularly well with the Aβ1–42:40 ratio in our study, TNF-α levels may be partly determined by this ratio. Higher levels of Aβ1–42 can promote the formation of toxic oligomers [57-59], and it therefore seems possible that the increased level of Aβ oligomers in PS1/APP mice (compared to APPsw) and the level of oligomeric forms present in the brains of our transgenic mice may be related to the amount of TNF-α being produced.

It is important to consider the nature of the exact form of Aβ that may be most responsible for the inflammatory events seen in AD brains. Aβ can exist in various forms (monomeric, dimeric, oligomeric and fibrillar), but it is not yet clear which of these forms are most potent in inducing inflammatory cellular responses [57,60,61]. This is of interest because the oligomeric forms of Aβ which are thought to be the most toxic are produced more readily by Aβ1–42 (for review see [62]). Future studies will assess the relative proportions of monomers/dimers, oligomers or fibrils occurring in these mice brains and their relationship with the cytokine increases observed.

## List of abbreviations

AD: Alzheimer's disease

APP: Amyloid precursor protein

APPsw: Amyloid precursor protein Swedish mutation

PS1: Presenilin 1

Aβ: Beta-amyloid

Tg: Transgenic

TNF: Tumor necrosis factor

IL-x: Interleukin-x

IL-1ra: Interleukin-1 receptor antagonist

GM-CSF: Granulocyte macrophage colony stimulating factor

PBS: Phosphate buffered saline

COX-2: Cyclo-oxygenase-2

PGE2: Prostaglandin E2

IFN: Interferon

NSAID: Non-steroidal anti-inflammatory drug

## Competing interests

The author(s) declare that they have no competing interests.

## Authors' contributions

NP carried out the in-vitro brain slice assays, processed brain tissues, performed the Bio-plex assay, ELISAs and drafted the manuscript. DP conceived the design of the study, carried out Bio-plex assays, performed statistical analyses and aided in manuscript preparation. VM analyzed data and constructed dendrograms. AQ aided in ELISA and Bio-plex assays and collected mouse brain tissues. FC oversees management of the mouse colonies. MM aided in manuscript preparation and gave critical analysis of the manuscript.

## Supplementary Material

Additional File 1 Correlation table of levels of different β-amyloid species with cytokines in transgenic mouse models of Alzheimer's disease. Click here for file
