# Supplementary material for: Inflammatory cytokine levels correlate with amyloid load in transgenic mouse models of Alzheimer's disease
Source: J Neuroinflammation. 2005 Mar 11;2:9. doi: 10.1186/1742-2094-2-9 (PMC555557; doi:10.1186/1742-2094-2-9)
Supplement: Additional File 1 — Correlation table of levels of different β-amyloid species with cytokines in transgenic mouse models of Alzheimer's disease. [file 1742-2094-2-9-S1.htm]

## Supplementary Material

## **Inflammatory cytokine levels correlate with amyloid load in transgenic mouse models of Alzheimer's Disease**

Nikunj
Patel, Daniel Paris, Venkatarajan Mathura, Amita Quadros, Fiona Crawford,
Michael Mullan.  
Roskamp Institute, 2040 Whitfield Avenue, Sarasota, FL34243,
USA.*Table 1. Correlation table of different Amyloid beta with
cytokines.*

|  |  |  |  |  |  |  |  |  |  |  |  |  |  |  |  |  |  |  |
| --- | --- | --- | --- | --- | --- | --- | --- | --- | --- | --- | --- | --- | --- | --- | --- | --- | --- | --- |
|  |  |  |  |  |  |  |  |  |  |  |  |  |  |  |  |  |  |  |
|  | Insol. Abeta 1-42 | Sol. Abeta1-42 | Insol. Abeta1-40 | Sol. Abeta1-40 | Insol. Abeta total | Sol. Abeta. total | Insol. Abeta ratio 42/40 | Sol.Abeta ratio 42/40 | GMCSF | INFG | TNFA | IL-1A | IL-1B | IL-2 | IL-3 | IL-6 | IL-10 | IL-12p40 |
| Insol. Abeta 1-42 | 1 | 0.856525 | 0.76862 | 0.862803 | 0.795362 | 0.870849 | -0.12008 | 0.407755 | 0.358072 | 0.700138 | 0.714872 | 0.425318 | 0.532935 | 0.813205 | 0.783827 | 0.322875 | 0.592137 | 0.691438 |
| Sol. Abeta1-42 | 0.856525 | 1 | 0.89163 | 0.941287 | 0.902292 | 0.966554 | -0.39761 | 0.558519 | 0.209933 | 0.45757 | 0.759024 | 0.637404 | 0.700951 | 0.836318 | 0.816055 | 0.244598 | 0.661888 | 0.849233 |
| Insol. Abeta1-40 | 0.76862 | 0.89163 | 1 | 0.858148 | 0.999079 | 0.876142 | -0.52781 | 0.466707 | 0.160787 | 0.313156 | 0.770639 | 0.701248 | 0.743488 | 0.812913 | 0.818777 | 0.231334 | 0.713135 | 0.820703 |
| Sol. Abeta1-40 | 0.862803 | 0.941287 | 0.858148 | 1 | 0.870989 | 0.996389 | -0.3511 | 0.302474 | 0.21178 | 0.443965 | 0.712843 | 0.686671 | 0.731969 | 0.850817 | 0.825563 | 0.190698 | 0.654499 | 0.929145 |
| Insol. Abeta total | 0.795362 | 0.902292 | 0.999079 | 0.870989 | 1 | 0.888578 | -0.50817 | 0.469567 | 0.176368 | 0.343686 | 0.778149 | 0.692977 | 0.740219 | 0.8248 | 0.828387 | 0.240851 | 0.71543 | 0.824014 |
| Sol. Abeta. total | 0.870849 | 0.966554 | 0.876142 | 0.996389 | 0.888578 | 1 | -0.36671 | 0.370245 | 0.213678 | 0.45234 | 0.73241 | 0.68194 | 0.732333 | 0.856661 | 0.832381 | 0.206381 | 0.663658 | 0.919412 |
| Insol. Abeta ratio 42/40 | -0.12008 | -0.39761 | -0.52781 | -0.3511 | -0.50817 | -0.36671 | 1 | -0.29778 | -0.10547 | 0.077061 | -0.37104 | -0.60095 | -0.54296 | -0.38203 | -0.40627 | -0.12225 | -0.47483 | -0.47124 |
| Sol.Abeta ratio 42/40 | 0.407755 | 0.558519 | 0.466707 | 0.302474 | 0.469567 | 0.370245 | -0.29778 | 1 | 0.311027 | 0.425221 | 0.590167 | 0.241367 | 0.303183 | 0.468537 | 0.497754 | 0.490114 | 0.552582 | 0.233584 |
| GMCSF | 0.358072 | 0.209933 | 0.160787 | 0.21178 | 0.176368 | 0.213678 | -0.10547 | 0.311027 | 1 | 0.725898 | 0.497545 | 0.216704 | 0.367022 | 0.405127 | 0.468272 | 0.894568 | 0.595558 | 0.08368 |
| INFG | 0.700138 | 0.45757 | 0.313156 | 0.443965 | 0.343686 | 0.45234 | 0.077061 | 0.425221 | 0.725898 | 1 | 0.6609 | 0.063889 | 0.253305 | 0.588328 | 0.5955 | 0.671523 | 0.513083 | 0.286104 |
| TNFA | 0.714872 | 0.759024 | 0.770639 | 0.712843 | 0.778149 | 0.73241 | -0.37104 | 0.590167 | 0.497545 | 0.6609 | 1 | 0.570155 | 0.707545 | 0.883098 | 0.915401 | 0.545499 | 0.828822 | 0.684771 |
| IL-1A | 0.425318 | 0.637404 | 0.701248 | 0.686671 | 0.692977 | 0.68194 | -0.60095 | 0.241367 | 0.216704 | 0.063889 | 0.570155 | 1 | 0.959891 | 0.666677 | 0.700383 | 0.147195 | 0.676642 | 0.78483 |
| IL-1B | 0.532935 | 0.700951 | 0.743488 | 0.731969 | 0.740219 | 0.732333 | -0.54296 | 0.303183 | 0.367022 | 0.253304 | 0.707545 | 0.959891 | 1 | 0.739412 | 0.783754 | 0.298254 | 0.762563 | 0.781629 |
| IL-2 | 0.813205 | 0.836318 | 0.812913 | 0.850817 | 0.8248 | 0.856661 | -0.38203 | 0.468537 | 0.405127 | 0.588328 | 0.883098 | 0.666677 | 0.739412 | 1 | 0.989708 | 0.422696 | 0.847465 | 0.775775 |
| IL-3 | 0.783827 | 0.816055 | 0.818777 | 0.825563 | 0.828387 | 0.832381 | -0.40627 | 0.497754 | 0.468272 | 0.5955 | 0.915401 | 0.700383 | 0.783754 | 0.989708 | 1 | 0.484267 | 0.895235 | 0.762973 |
| IL-6 | 0.322875 | 0.244598 | 0.231334 | 0.190698 | 0.240851 | 0.206381 | -0.12225 | 0.490114 | 0.894568 | 0.671523 | 0.545499 | 0.147195 | 0.298254 | 0.422696 | 0.484267 | 1 | 0.678478 | 0.058561 |
| IL-10 | 0.592137 | 0.661888 | 0.713135 | 0.654499 | 0.71543 | 0.663658 | -0.47483 | 0.552582 | 0.595558 | 0.513082 | 0.828822 | 0.676642 | 0.762563 | 0.847465 | 0.895235 | 0.678477 | 1 | 0.5877 |
| IL-12p40 | 0.691438 | 0.849233 | 0.820703 | 0.929145 | 0.824014 | 0.919412 | -0.47124 | 0.233584 | 0.08368 | 0.286104 | 0.684771 | 0.78483 | 0.781629 | 0.775775 | 0.762973 | 0.058561 | 0.5877 | 1 |
|  |  |  |  |  |  |  |  |  |  |  |  |  |  |  |  |  |  |  |
| df=24 |  | | | | | | | | | | | | | | | | | |
| 1% significance is |r|>=0.496 | |  | | | | | | | | | | | | | | | | |
| 5% significance is |r|>=0.388 | |  | | | | | | | | | | | | | | | | |
|  |  |  |  |  |  |  |  |  |  |  |  |  |  |  |  |  |  |  |
